# Long-term durable repaired cartilage induced by SOX9 *in situ* with bone marrow-derived mesenchymal stem cells

**DOI:** 10.7150/ijms.52510

**Published:** 2021-01-26

**Authors:** Xiaowei Zhang, Shili Wu, Yong Zhu, Cong-Qiu Chu

**Affiliations:** 1 Division of Arthritis and Rheumatic Diseases, Oregon Health & Science University, Portland, Oregon 97239.; 2 Section of Rheumatology, VA Portland Health Care System, Portland, Oregon 97239.; 3 Vivoscript, Inc, P. O. Box 63025, Irvine, CA 92602.

**Keywords:** cartilage repair, collagen membrane, proteoglycan, osteoarthritis

## Abstract

**Background:** Microfracture is a common procedure for cartilage repair, but it often produces inferior fibrocartilage. We previously reported that a super positively charged SOX9 (scSOX9) promoted hyaline-like cartilage regeneration by inducing bone marrow derived mesenchymal stem cell differentiation into chondrocytes *in vivo*. Here we examined the long-term efficacy of cartilage repair induced by microfracture with scSOX9 by assessing the biomechanical property of the repaired cartilage.

**Methods:** A cartilage defect was created at the right femoral trochlear groove in New Zealand female rabbits and microfracture was performed. The scSOX9 protein was administered at the site of microfracture incorporated in a collagen membrane.

**Results:** At 12 and 24 weeks, scSOX9 treatment induced hyaline-like cartilage while collagen-membrane alone induced fibrocartilage and mutant scSOX9-A76E poorly induced cartilage repair. The cartilage matrix in scSOX9-treated group showed highly enriched proteoglycan content. Consistent with the histological feature and the thickness of the repaired cartilage, the mechanical property of scSOX9-induced cartilage was also similar to that of normal cartilage.

**Conclusion:** This long-term *in vivo* study demonstrated that in combination with microfracture, scSOX9 was able to induce reparative tissue with features of hyaline cartilage which was durable in long-term. This technology has the potential to translate into clinical use for cartilage repair to prevent progression to osteoarthritis.

## Introduction

Quality articular cartilage repair remains an unmet need for treatment of cartilage injury to prevent progression into osteoarthritis (OA) 1, 2. Stimulating regeneration of articular cartilage by mobilizing bone marrow mesenchymal stem cells (MSCs) with microfracture has been a common procedure for treating cartilage injury in clinical practice in the expectation that hyaline cartilage will be regenerated. In reality, microfracture induces biomechanically inferior fibrocartilage or fibro-hyaline hybrid tissue, fails to provide a long-term therapeutic effect, and in some cases it even results in deterioration of the joint function and leads to progression into OA 3, 4.

In a rabbit cartilage injury model, we modified the microfracture procedure by introducing a chondrogenic transcription factor, SRY-type high-mobility group box 9 (SOX9) (in the form of super positively charged recombinant protein, scSOX9) at the site of microfracture. In this modified microfracture procedure, scSOX9 induced hyaline-like cartilage generation in a short-term observation 5. Here we report that in a long-term observation, scSOX9-induced reparative cartilage was durable and its biomechanical property was superior to that from microfracture only treatment.

We envision that scSOX9 would be an agent to explore for regeneration of articular cartilage to slow down the progression of OA.

## Materials and methods

The study was approved by Office of Research and Development, VA Portland Health Care System (VAPORHCS), Portland, Oregon, USA. All methods were performed in accordance with the guidelines and regulations of the National Institutes of Health and the VAPORHCS.

### Reagents

The scSOX9 and scSOX9-A76E (a muted scSOX9) recombinant proteins were generated by Vivoscript, Inc. (Irvine, CA) and were incorporated onto collagen membrane as described 5, 6. Briefly, a fusion protein is constructed, with a super positively charged green fluorescent protein (scGFP) moiety on the N-terminus, followed with the human SOX9 protein moiety and a C-terminal polyarginine tag (11R). This recombinant protein, referred as supercharged SOX9 (scSOX9), was expressed in E. coli and purified through inclusion body extraction and protein refolding. The mutant scSOX9-A76E was generated by substituting alanine at position 76 of the SOX9 moiety with glutamine, resulting in the loss of the chondrogenic property of SOX9.

### Animals and surgery

All animal studies were approved by Institutional Animal Care and Use Committee of the VAPORHCS, #3469-15). Mature female New Zealand white rabbits with body weight of 3.5-4.0 kg were included in the study. A cylindrical osteochondral defect of 4 mm in diameter and 3 mm in depth is created at the patella groove of femur as described 5,7,8. Microfracture was performed using a 0.9 mm Kirschner wire tapped into the subchondral bone to a depth of approximately 3 mm until bleeding from the hole is apparent 5. Three microfracture holes in a triangular configuration were created within each full thickness chondral defect. The defect of cartilage was covered with either scSOX9 or scSOX9-A76E bound collagen membrane, or membrane only 5. Rabbits were set for free in movement in their home cage.

### Gross and histological assessment

At 12 and 24 weeks post-surgery, the distal femur was extirpated and examined and photographed for evaluation in a blinded manner according to the International Cartilage Repair Society (ICRS) macroscopic assessment scale for cartilage repair as described 5. Histological analysis of cartilage repair was scored according to the ICRS recommendation. The stained sections were coded for their *in vivo* treatment by an independent laboratory technician and the coded sections were scored by two investigators (XZ and CQC) 5.

### Biomechanics of repaired cartilage

The distal femur was examined by three-dimensional assessment for repaired cartilage and bone structure to gain global biomechanical features by using the Mach-1 V500c mechanical tester (Biomomentum). At each site, a rapid indentation of the cartilage was performed in 1 second to a depth of 50 μm using a spherical indenter (diameter = 1.0 mm) attached under a single-axis load cell (100 N load range and resolution of 5 mN). The load versus displacement curve obtained in indentation fitted to an elastic model in indentation with corresponding cartilage thickness for determination of mechanical properties. To measure cartilage thickness, the spherical indenter was replaced with a needle probe and the mechanical tester moves the needle towards the sample. The cartilage thickness corresponds to the displacement between the first increase in the load (cartilage surface) and a steep increase in the load (subchondral bone) 9.

### Statistics

Gross and histological scores and biomechanical analysis were analyzed with one-way ANOVA followed by post hoc analysis with Newman-Keuls Multiple Comparison Test. Differences of p<0.05 were considered statistically significant.

## Results

### Gross morphology of cartilage repair

**Figure 1:**
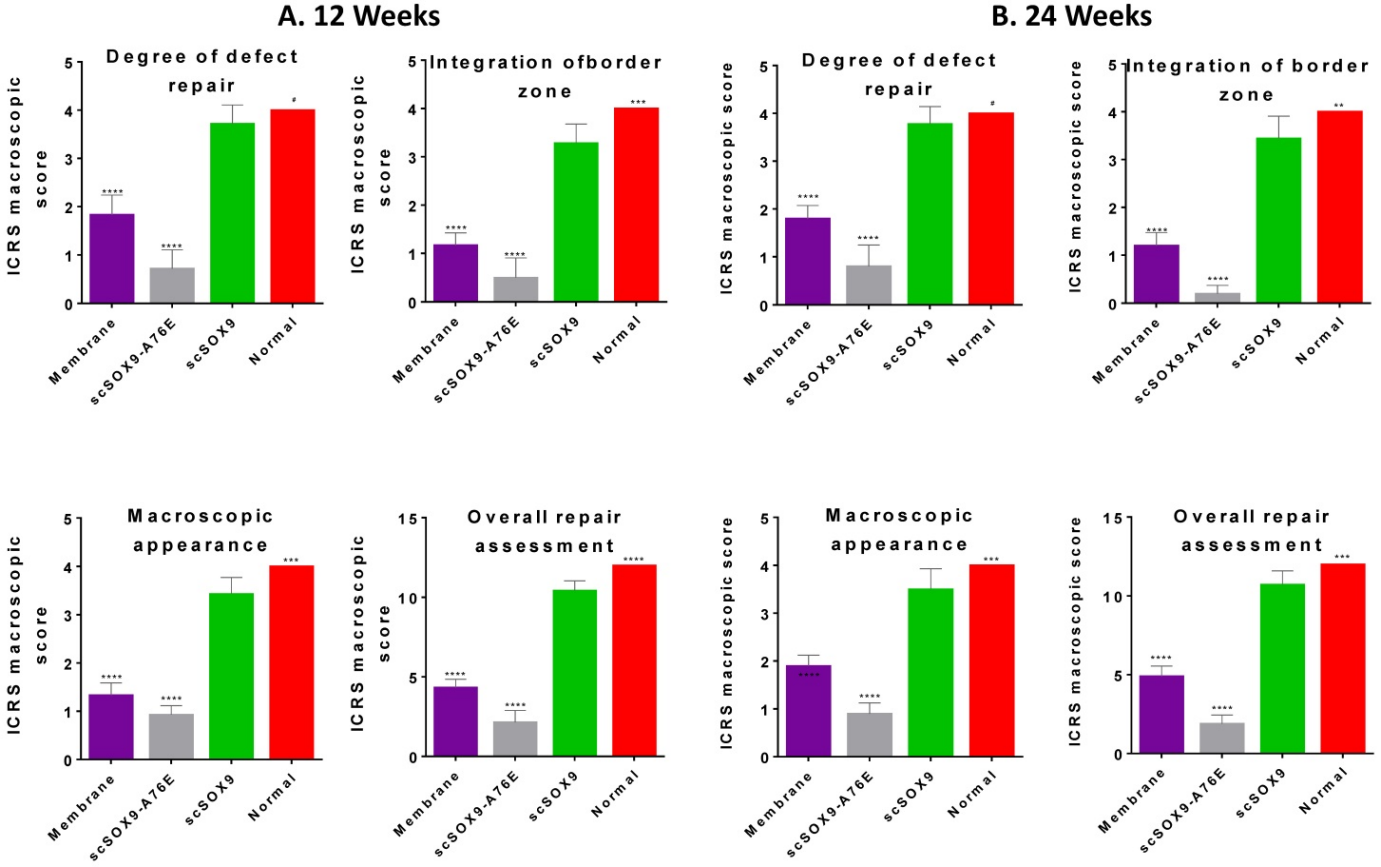
** Macroscopic assessment of cartilage repair.** At 12 (A) and 24 (B) weeks after microfracture treatment, the distal femurs of rabbits were examined grossly in comparison with normal cartilage surface of the distal femur as reference. Macroscopic assessment of cartilage repair was quantified by International Cartilage Repair Society (ICRS) macroscopic evaluation scale. Results were presented as the mean ± SD; n = 8 in membrane only and n = 12 in scSOX9 and scSOX9-A76E group. *p < 0.05; ** p < 0.01; *** p < 0.001; **** p < 0.0001; # Not significant. Membrane: microfracture and collagen membrane; scSOX9-A76E: microfracture and collagen membrane containing mutant form of super-charged SRY-type high-mobility group box-9 (scSOX9-A76E); scSOX9: microfracture and collagen membrane containing scSOX9.

The gross morphology of repaired tissue was compared with the same area of the contralateral knee using ICRS macroscopic assessment scale that consists of defect repair, integration to border zone and macroscopic appearance. Defects in the group treated with microfracture with collagen membrane alone had better coverage than those in the group with microfracture plus scSOX9-A76E. In contrast, in each of animals treated with microfracture plus scSOX9, the entire original defect was filled with glossy semitransparent repaired tissue that appeared to be smooth and well-integrated with the surrounding tissues at both 12 and 24 weeks (**Figure 1**). The higher total ICRS scores were seen in microfracture plus scSOX9 treated group than any other groups indicating that scSOX9 significantly improved cartilage regeneration.

### Histology of cartilage repair

**Figure 2:**
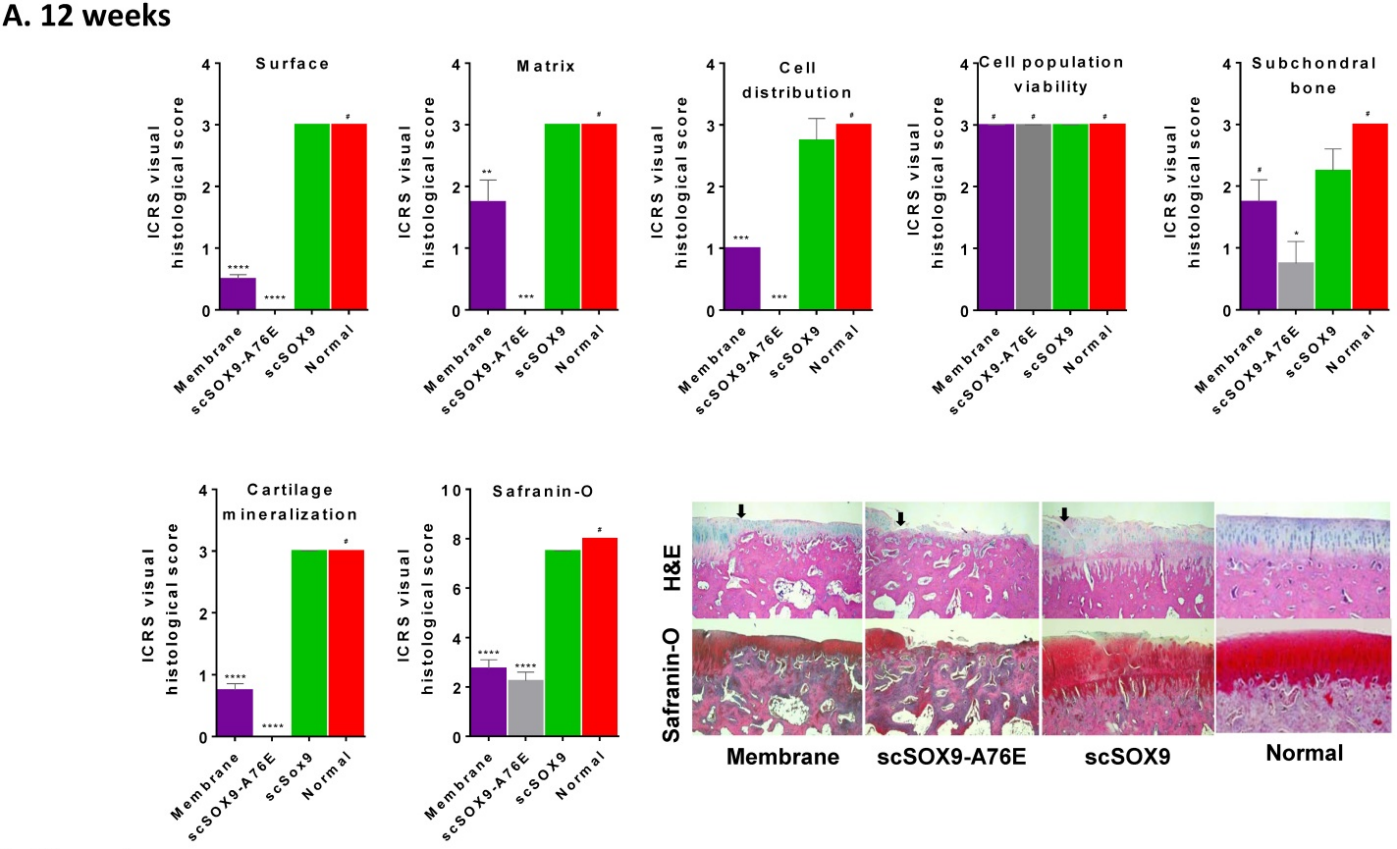

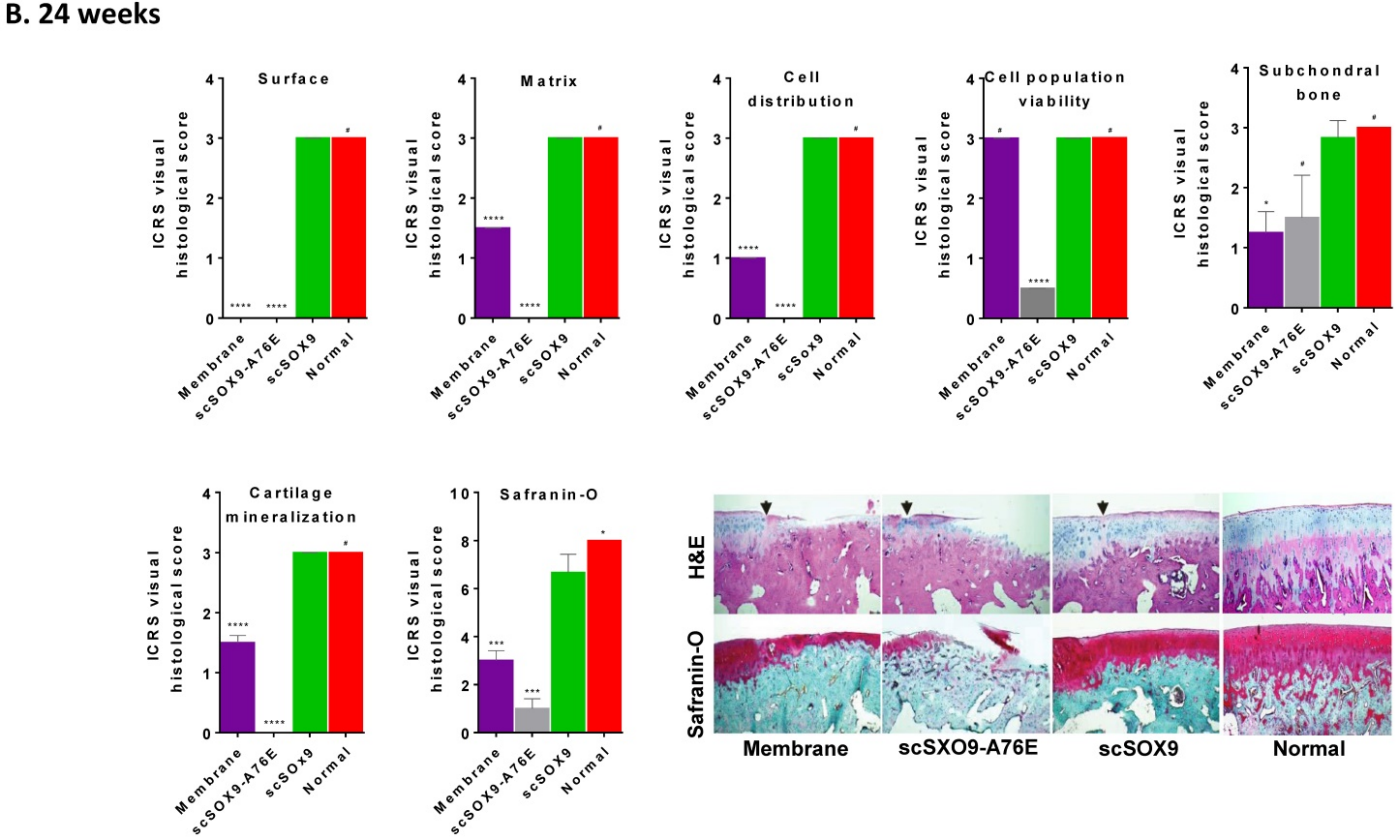
** Histological assessment of cartilage repair.** The distal femurs were fixed in 10% formalin, decalcified, embedded in paraffin, and cut into 5 µm sections. Sections from each sample were then stained with hematoxylin and eosin (H&E) for morphological evaluation. Safranin-O and fast green staining was used to assess matrix proteoglycan distribution. The black arrow indicates the defect boundaries. H&E staining sections were scored according to ICRS assessment system updated recommendations. Each of the categories of assessment was reported separately as recommended by ICRS. Quantification of Safranin-O staining was assessed as described previously 5. Results were presented as the mean ± SD; n = 8 in membrane only and n = 12 in scSOX9 and scSOX9-A76E group. *p < 0.05; **p < 0.01; *** p < 0.001; ****p < 0.0001; # Not significant. Membrane: microfracture and collagen membrane only; scSOX9-A76E: microfracture and collagen membrane containing scSOX9-A76E; scSOX9: microfracture and collagen membrane containing scSOX9. Normal: normal cartilage taken from the contralateral femur of the same region as in the cartilage defect and repaired region of the experiment.

As shown in **Figure 2**, scSOX9 treated group induced hyaline-like cartilage while collagen-membrane only induced fibrocartilage and mutant scSOX9-A76E poorly induced cartilage repair. A thin layer of fibrocartilage/hyaline-like cartilage repair in collagen membrane only group is evident, while un-repaired subchondral bone or calcified cartilage were seen in scSOX9-A76E treated group. In contrast, a thick layer of high-quality hyaline-like cartilage was shown in the scSOX9-treated group. As seen at 8 weeks 5, there was no difference found between treatment groups in cell viability in reparative cartilage tissue at 12 or 24 weeks. The cartilage matrix in scSOX9-treated group showed highly enriched proteoglycan content as indicated by dense Safranin-O staining at both 12 and 24 weeks (**Figure 2A and B**). These observations confirmed that scSOX9-induced cartilage repair was endurable in long term.

### Mechanical property of repaired cartilage

**Figure 3:**
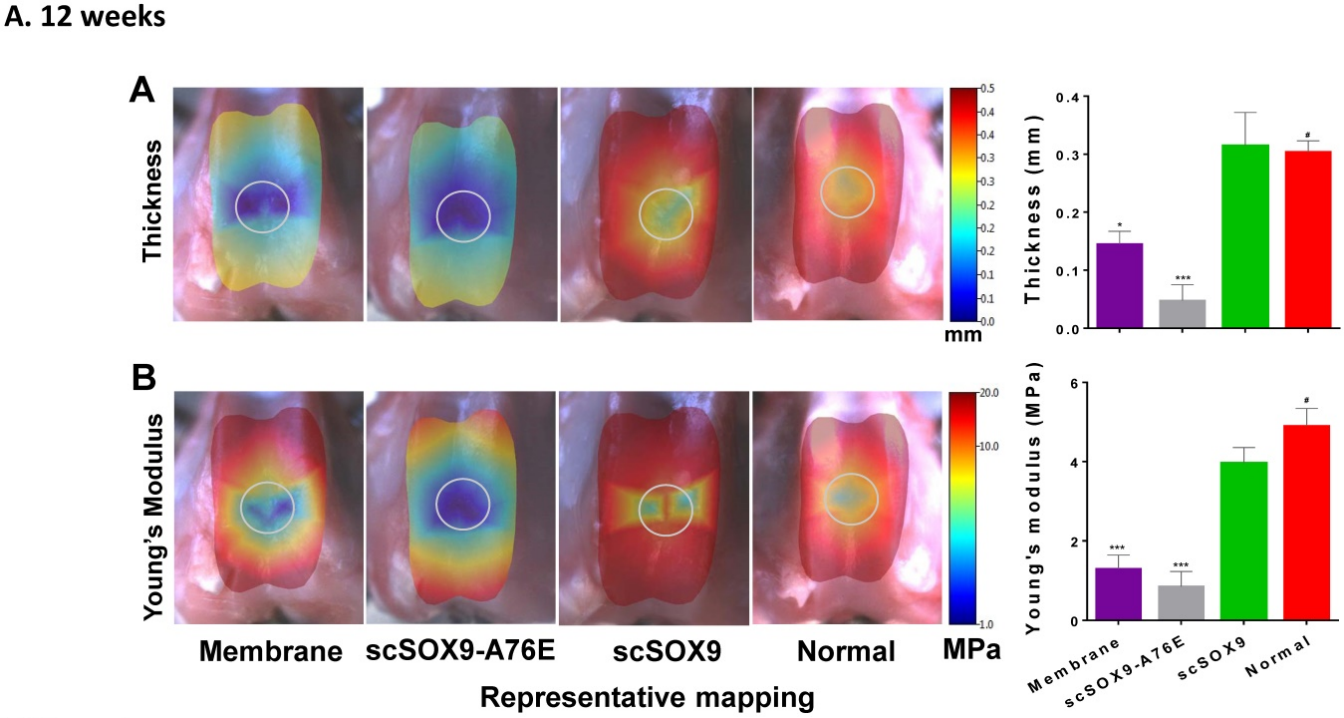

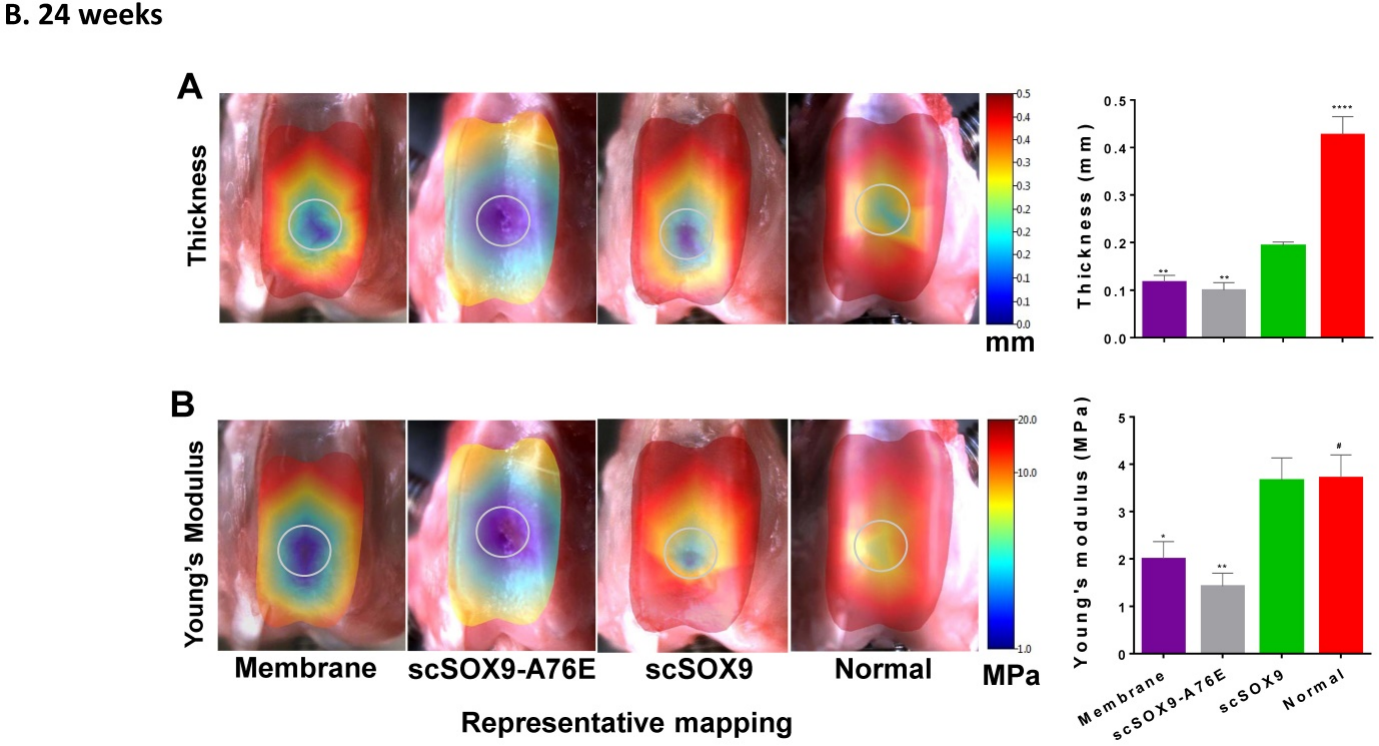
** Mechanical property of repaired cartilage.** Biomechanical property of repaired cartilage (right distal femur) was measured by indentation and thickness of cartilage were obtained through a needle technique. The left distal femur from rabbits was used as normal samples for comparison. The macroscopic graph shows representative mapping for measurement and the circle indicates original cartilage defect in each sample. Mechanical Young's modulus at 50 µm indentation amplitude and cartilage thickness are shown in bar graphs for comparison between control and treated group. Data are presented for each region as the averaged of all control (n = 8) and all treated groups (n = 12), *p<0.05; ** p<0.01; *** p<0.001; # Not significant.

The mechanical property of repaired cartilage was tested by using the Biomomentum system as described by Sim et al. 9. The thickness of repaired cartilage induced by scSOx9 was comparable to that of normal cartilage in the same area at 12 weeks and at 24 weeks, whereas collagen membrane only induced a thinner layer of cartilage, and scSOX9-A76E treated group showed even worse repaired tissue (**Figure 3A and B**). Consistent with the histological data, the mechanical property of scSOX9 induced cartilage was also similar to that of normal cartilage at both 12 (**Figure 3A**) and 24 weeks (**Figure 3B**).

## Discussion

Regeneration of articular cartilage *in situ* is an ultimate goal for cartilage repair. Microfracture attempts to reach this goal by mobilizing MSCs from the bone marrow. However, the clinical outcome in cartilage repair by microfracture failed to achieve the goal 3,4. By introducing scSOX9 at the time of microfracture, we improved the outcome of cartilage repair as judged by macroscopic and histological assessment in a short-term (8 weeks) observation in a rabbit cartilage injury model 5. We continued to observe the similar therapeutic effect by scSOX9 for 12 weeks, then extended the duration further to 24 weeks after the treatment. The results at 24 weeks were similar to those observed at 12 weeks and demonstrated a long-term efficacy of scSOX9 transduction for cartilage repair. We showed that *in vitro* scSOX9 was able to stimulate bone marrow derived MSCs differentiation into chondrocytes 5. It is likely that the scSOX9 protein enters into the MSCs contained in the bone marrow at the microfracture site and then turn them into chondrocytes for cartilage regeneration to cover the cartilage defect.

Assessment of clinical outcomes in animal models for cartilage repair has been hampered due to lack of instruments for measuring clinical signs reliably. Here we used a surrogate to measure the quality of repaired cartilage - biomechanical property. Previous studies measuring cartilage biomechanical features involve separation of cartilage layer that are destructive 2. Measurement of isolated cartilage suffers being destructive and inaccuracy in reflection of the property of articular cartilage in its native setting in the joint. The technique employed in our study is a desirable measurement since it is nondestructive and measures cartilage biomechanical property with a subchondral bone as a single unit 9. Indentation with a force to the intact cartilage surface in fresh tissue simulates *in vivo* situation 2,9. It has been shown that indentation measurement of articular cartilage correlates with histological scores and biomechanical properties. The measured electromechanical quantitative parameters are strongly related to the structure and organization of the collagen network and content of matrix protein such as proteoglycan 2,9. Our results at both 12 and 24 weeks confirmed this correlation. It must be noted that the thickness of repaired cartilage appeared thinner at 24 weeks than at 12 weeks. This may suggest that the regenerated cartilage is not as durable as the natural, intact cartilage and there is room for improvement for our scSOX9-based approach. This may also be due to aging of the animals. However, there is no significant change in biomechanical property based on our indentation tests. Obviously, a longer-term observation is required to further address this issue.

Our study has limitations. The cartilage injury and repaired area is not considered weight bearing one and cartilage injury is acute and does not well represent the clinical situation. These can be addressed by future studies in treating cartilage injury in weight bearing region of the joint and in aged injury. The latter is more relevant to clinical situation and the results will be more informative for our ongoing studies to test whether scSOX9 intraarticular injection will be effective in treating post-traumatic OA in murine models. Some safety aspects of this type of therapy will need to be addressed in even longer-term studies. For example, intensifying chondrogenic differentiation of stem cells can predispose differentiated chondrocyte-like cells to early hypertrophy. SOX9 is known to repress chondrocyte hypertrophy and inhibit osteogenesis concomitantly to promote chondrogeneis 10,11. We have not observed hypertrophy or ossification up to 24 weeks after treatment with scSOX9. Much more is to be learnt at molecular level about MSC differentiation in such *in vivo* studies. We are performing gene expression assay at different time points during the MSC differentiation and cartilage repair process to dissect what other factors are involved.

In OA joints, the number of chondrocyte progenitors and MSCs is increased but they failed to repair the degraded cartilage 12,13. It was found that OA chondrocytes have decreased levels of SOX9 14. *In vitro* studies have shown that viral vector mediated SOX9 gene delivery could restore OA chondrocyte function to secrete collagen type II and proteoglycan 15,16. Therefore, intraarticular delivery of scSOX9 could be a viable therapeutic approach to cartilage regeneration for patients with OA.

## References

[1] Huey DJ, Hu JC, Athanasiou KA (2012). Unlike bone, cartilage regeneration remains elusive. Science.

[2] Maglio M, Brogini S, Pagani S, Giavaresi G, Tschon M (2019). Current Trends in the Evaluation of Osteochondral Lesion Treatments: Histology, Histomorphometry, and Biomechanics in Preclinical Models. Biomed Res Int.

[3] Gao L, Goebel LKH, Orth P, Cucchiarini M, Madry H (2018). Subchondral drilling for articular cartilage repair: a systematic review of translational research. Dis Model Mech.

[4] Mithoefer K, McAdams T, Williams RJ, Kreuz PC, Mandelbaum BR (2009). Clinical efficacy of the microfracture technique for articular cartilage repair in the knee: an evidence-based systematic analysis. Am J Sports Med.

[5] Zhang X, Wu S, Naccarato T, Prakash-Damani M, Chou Y, Chu CQ (2017). Regeneration of hyaline-like cartilage *in situ* with SOX9 stimulation of bone marrow-derived mesenchymal stem cells. PLoS One.

[6] Zhou H, Wu S, Joo JY, Zhu S, Han DW, Lin T (2009). Generation of induced pluripotent stem cells using recombinant proteins. Cell Stem Cell.

[7] Yang HS, La WG, Bhang SH, Kim HJ, Im GI, Lee H (2011). Hyaline cartilage regeneration by combined therapy of microfracture and long-term bone morphogenetic protein-2 delivery. Tissue engineering Part A.

[8] Strauss E, Schachter A, Frenkel S, Rosen J (2009). The efficacy of intra-articular hyaluronan injection after the microfracture technique for the treatment of articular cartilage lesions. The American journal of sports medicine.

[9] Sim S, Chevrier A, Garon M, Quenneville E, Yaroshinsky A, Hoemann CD (2014). Non-destructive electromechanical assessment (Arthro-BST) of human articular cartilage correlates with histological scores and biomechanical properties. Osteoarthritis Cartilage.

[10] Leung VY, Gao B, Leung KK, Melhado IG, Wynn SL, Au TY (2011). SOX9 governs differentiation stage-specific gene expression in growth plate chondrocytes via direct concomitant transactivation and repression. PLoS Genet.

[11] Cao L, Yang F, Liu G, Yu D, Li H, Fan Q (2011). The promotion of cartilage defect repair using adenovirus mediated Sox9 gene transfer of rabbit bone marrow mesenchymal stem cells. Biomaterials.

[12] Fellows CR, Williams R, Davies IR, Gohil K, Baird DM, Fairclough J (2017). Characterisation of a divergent progenitor cell sub-populations in human osteoarthritic cartilage: the role of telomere erosion and replicative senescence. Sci Rep.

[13] Campbell TM, Churchman SM, Gomez A, McGonagle D, Conaghan PG, Ponchel F (2016). Mesenchymal Stem Cell Alterations in Bone Marrow Lesions in Patients With Hip Osteoarthritis. Arthritis Rheumatol.

[14] Cucchiarini M, Thurn T, Weimer A, Kohn D, Terwilliger EF, Madry H (2007). Restoration of the extracellular matrix in human osteoarthritic articular cartilage by overexpression of the transcription factor SOX9. Arthritis Rheum.

[15] Li Y, Tew SR, Russell AM, Gonzalez KR, Hardingham TE, Hawkins RE (2004). Transduction of passaged human articular chondrocytes with adenoviral, retroviral, and lentiviral vectors and the effects of enhanced expression of SOX9. Tissue Eng.

[16] Tew SR, Li Y, Pothacharoen P, Tweats LM, Hawkins RE, Hardingham TE (2005). Retroviral transduction with SOX9 enhances re-expression of the chondrocyte phenotype in passaged osteoarthritic human articular chondrocytes. Osteoarthritis Cartilage.

